# Windows into the past: recent scientific techniques in dental analysis

**DOI:** 10.1038/s41415-024-7053-0

**Published:** 2024-02-09

**Authors:** Roger Forshaw

**Affiliations:** KNH Centre for Biomedical Egyptology, Faculty of Biology, Medicine and Health, Stopford Building, Oxford Road, University of Manchester, Manchester, M13 9PL, UK

## Abstract

Teeth are the hardest and most chemically stable tissues in the body, are well-preserved in archaeological remains and, being resistant to decomposition in the soil, survive long after their supporting structures have deteriorated. It has long been recognised that visual and radiographic examination of teeth can provide considerable information relating to the lifestyle of an individual. This paper examines the latest scientific approaches that have become available to investigate recent and ancient teeth. These techniques include DNA analysis, which can be used to determine the sex of an individual, indicate familial relationships, study population movements, provide phylogenetic information and identify the presence of disease pathogens. A stable isotopic approach can shed light on aspects of diet and mobility and even research climate change. Proteomic analysis of ancient dental calculus can reveal specific information about individual diets. Synchrotron microcomputed tomography is a non-invasive technique which can be used to visualise physiological impactful events, such as parturition, menopause and diseases in cementum microstructure - these being displayed as aberrant growth lines.

## Key points

- Emphasises the importance of the investigation of ancient teeth to the study of anthropology, archaeology and forensics.
- Discusses the wide range of analytical techniques that are now available to investigate recent and ancient teeth.
- Highlights the extent of the information that can be unlocked from teeth and dental calculus.

## Introduction

Today, monitoring oral health provides key indicators relating to dental health, overall health and wellbeing, and can help to address patients' concerns involving their orofacial structures. Similarly, examining ancient dentitions can be fundamental in understanding the lives of our ancestors and can help to unlock the past. Teeth provide a window into the lifestyle of ancient peoples in a way that artefacts and historical sources of information do not.

Teeth, being highly mineralised, are sometimes the sole surviving human remains belonging to past individuals and the societies to which they belonged. Teeth are the only element of the skeleton coming into direct contact with the environment and being composed of dynamic tissues, they provide an extensive resource related to previous and present lifestyles. They can provide data relating to physiology, adaptation, evolution, genetic variation, social and cultural behaviour, diet, migration, growth and development, oral and general health, and identification in forensic contexts.^1^

Ancient teeth have been the subject of dental pathological investigations for many years, with some early published reports in this field being those of Mummery in 1870^2^ and Ruffer^3^ in 1920. During the twentieth century, there were major strides in the understanding of the anatomy, growth, physiology and diseases of the teeth. However, more recently, advanced scientific techniques have been applied to the study of ancient teeth. In 2014, this author published the paper ‘Dental indicators of ancient dietary patterns: dental analysis in archaeology',^4^ which discussed the wide range of analytical techniques that were then currently available to investigate ancient teeth. Now, ten years on, further advanced techniques and approaches have become available, while some of the existing methodologies have been refined and improved; these topics are the subject of this paper.

Different growth layers of the tooth can be utilised in dental analysis, the choice of which depending on their particular characteristics, such as the frequency with which new layers are added, the length of time in which this process occurs and for how long the layers remain unchanged. Such parameters determine the suitability of each dental tissue to answer a particular research question.^5^ For example, enamel is useful in a minimally destructive technique for biological sex determination; the soft tissue of the pulp is particularly suited for molecular analysis due to its being protected within the pulp chamber; cementum is a particular rich source of DNA; enamel and dentine are useful in stable isotope analysis as they are more resistant to diagenesis than bone; and dental calculus, from which it is possible to analyse preserved ancient biomolecules and dietary microfossils, is not an inherent part of the skeleton but a secondary material.

## Biological sex determination using amelogenin proteins in teeth

The determination of biological sex from the skeleton is fundamental in the study of past human populations and for establishing human identity in forensic contexts, such as mass disasters and war graves. Sex determination relies on two methods: the analysis of sexually dimorphic osteological features and the detection of DNA markers that are specific to the X and Y chromosome.^6^^,^^7^^,^^8^ However, biological sex can be difficult or impossible to assess from remains that are degraded by environmental taphonomic processes, both in respect of assessing osteological features and ancient DNA (aDNA) sequencing, a technique that requires good DNA survival. Another problem can be the reliable estimation of sex in the subadult skeleton as osteological traits are not sufficiently well-developed in the immature skeleton.^9^

An alternative method - utilising the enamel amelogenin proteins (proteins which play an important role in the mineralisation of enamel) - has been described by a number of researchers.^10^^,^^11^^,^^12^ Proteins are more robust than DNA molecules, particularly when they are physically associated with mineral surfaces such as enamel, and so have a greater potential to persist in the archaeological record. In this process, enamel is sampled by the minimally destructive method of acid etching the surface of the tooth and the enamel residue analysed by liquid chromatography and mass spectrometry. The sex chromosome-linked isoforms of amelogenin are subsequently able to be identified.

Parker and co-workers^13^ recently applied this technique, but rather than acid etching the surface, they sampled enamel sections from the teeth, a process which maximised the extraction of sexually dimorphic peptides from the amelogenin protein, although the method is more destructive to the tooth morphology. However, they determined that a subset of low signal male samples could be falsely assigned as female. Accordingly, in forensic cases where correct identification is essential, DNA analysis may be the technique of choice as additional Y chromosome markers can be included in the testing regime.

Other similar methods to determine biological sex from peptides in tooth enamel have recently been described, again based on liquid chromatography and high-resolution mass spectrometry.^14^^,^^15^^,^^16^ Although good results have been obtained utilising these methods, the times required for the analyses are quite lengthy and due to their high cost, many laboratories do not have access to the type of necessary instrumentation.

Particularly in archaeological contexts, the minimally destructive acid etch technique does have a number of advantages. The gross anatomical features of the tooth are maintained. The process can be applied just as readily to deciduous or permanent teeth and to skeletons with missing, degraded or ambiguous osteological sex markers. In addition, the process is rapid and relatively inexpensive compared to DNA sequencing.

## Estimating age at death utilising carbon-14

If a large number of victims are killed in mass disasters, accidents or terrorist attacks, a top priority is to confirm their identity, a procedure that involves fingerprinting, dental findings and DNA typing. These investigations can be problematic when the human remains are in poor condition or the elapsed post-mortem time is lengthy. An important factor is to determine the age of the deceased and this is usually estimated by means of age-related changes in the skeleton. However, in applying this criteria, the estimated age range can vary by up to ten years, which is not precise enough when trying to match unidentified bodies/remains to missing persons lists. Similarly, the method of age assessment by evaluating occlusal tooth wear will not provide a sufficiently accurate age determination.

More recently, radiocarbon, or carbon-14 (^14^C), present in tooth enamel as a result of nuclear bomb testing during the cold war, has been found to be a remarkably accurate indicator of when a person is born.^17^^,^^18^^,^^19^ The concentration of natural ^14^C in the atmosphere in relation to all the carbon has been fairly constant in the region of 1.2 parts per trillion over the past several millennia. However, atmospheric detonations of nuclear weapons during the 1950s and early 1960s doubled the concentration of the ^14^C in the atmosphere. With the banning of atmospheric nuclear testing by the Partial Nuclear Test Ban Treaty in 1963, these levels began to decrease. Data showing ^14^C concentration in the atmosphere as a function of time have been established.

**Fig. 1:**
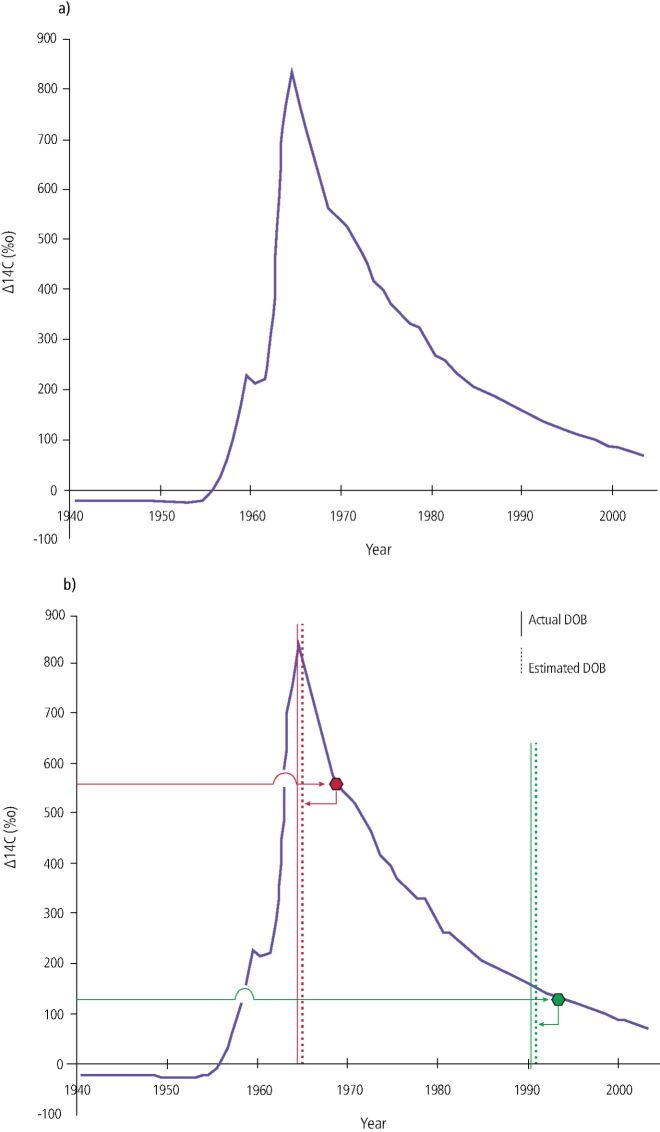
Dating: northern hemisphere atmospheric ^14^C concentration as a function of time. a) This graph shows the northern hemisphere growing season average of atmospheric ^14^C concentration in CO_2_ from 1940-2007. It is constructed using several different datasets that used tree rings, unpublished recent plant growth and direct atmospheric sampling to provide carbon samples. b) To estimate an individual's date of birth, the level of ^14^C measured in tooth enamel is plotted on to the curve of atmospheric ^14^C against time (blue) to find the year of enamel synthesis (right-pointing arrows). The known age at enamel formation for individual teeth is then subtracted from the year obtained to give the date of birth (left-pointing arrows, dashed vertical lines). Two representative cases are shown; two teeth were analysed for the case depicted in red and one tooth for the example shown in green. Solid vertical lines represent the actual date of birth. Reproduced with permission from Bucholz *et al*., ‘Year of birth determination using radiocarbon dating of dental enamel', *Surface and Interface Analysis*, 2009, Wiley^18^

^14^C accumulates in tooth enamel while the enamel is being formed in childhood, and as there is no turnover of enamel during life, then the ^14^C concentration of the enamel reflects the level in food sources at the time of enamel formation. By determining the amount of ^14^C in an enamel sample using accelerator mass spectrometry, it is possible to assess an individual's date of birth from a graph which charts the known level of atmospheric ^14^C over time. This will then establish the year that the enamel began to develop and then the number of years for the enamel to form is deducted from the year obtained from the graph. This gives an estimated date of birth, a figure that is considered to be accurate to ± 1.5 years (Fig. 1). The dating technique will not work for people born before 1943, as all their teeth would have been formed before atmospheric nuclear testing began in 1955.

## Stable isotope analysis

Stable isotope analysis is a well-established technique in archaeology for exploring diet and mobility in past populations^4^^,^^20^ and can even be applied to ancient fossil remains.^21^^,^^22^^,^^23^ Stable isotope investigations are based on the principle that human and animal body tissues will reflect the isotopic composition of food and water ingested during their lifetimes - ‘you are what you eat'. Isotopes are different forms of the same element and it is the assessment of the characteristic patterns or isotope signatures that are utilised in this technique.^24^^,^^25^^,^^26^

Although bone is often the tissue of choice in these studies due to its higher concentration of collagen, bone hydroxyapatite is vulnerable to post-depositional changes in an archaeological setting. Because of the excellent preservation of biogenic elements in the tooth structure and their resistance to breakdown and decay, teeth are frequently used in stable isotope analysis. As the enamel of the teeth is formed during infancy and childhood, and after which its chemical composition does not change, then the structure will reflect the diet and locality of an individual during the period of enamel formation.^27^

Both nitrogen and carbon stable isotope analyses of serial sections of first molar dentine collagen can capture dietary changes early in life, for example during weaning.^28^ Similarly, third molar teeth are also useful as they begin to form at the dentine-enamel junction between 7-10 years of age and then continue growing through 18-25 years, when the apical root tip is completed. By analysing levels of carbon and nitrogen isotopes in serial microsections of these teeth, it is possible to reconstruct dietary changes across small windows of time, typically 0.5-2 years. For example, Eerkens and colleagues^29^ analysed stable isotope data from microsections of a number of individuals from a Late Holocene hunter-gatherer site (c. 500 BC - AD 800) in central California (CA-SOL-11). Their research not only demonstrated a considerable dietary variation between the individuals, but also that some of the hunter-gatherers significantly changed diet during the course of their lives, suggesting movements between different sources of food and possibly their resettlement. These types of data can be helpful in providing information on inter- and intra-individual variation in diets over periods of time.

Teeth are also a useful indicator of past environments, including changes in climatic conditions. With record-breaking heat waves and unprecedented floods wreaking havoc, climate change is an extremely important issue in today's world. It is also recognised as a significant factor throughout human history, causing famine, migrations and disruption to political systems.

Climate change can be tracked by variations in oxygen isotope levels in teeth, the ratios of the lighter ^16^O isotope and the heavier ^18^O isotope, the measure of which is known as δ^18^O. These changes take place during evaporation and condensation of humid air masses. Oxygen isotope ratios of human body tissue are strongly related to the composition of water that is drunk during life. Consequentially, changes in the isotope values of the teeth and bones of peoples living through different time periods and for a given locality will provide an indicator of any past variations in climate.

A study was carried out by Touzeau and co-workers^30^ on a group of 48 ancient Egyptian mummies housed in museums in Lyon, France, many of which originated from ancient Thebes. Based on archaeological evidence, they had been assigned dates varying from about 5500 BC to 2500 BC. Ground samples of tooth enamel were isotopically analysed and the results demonstrated an increase in oxygen isotope values (δ^18^O) of approximately 3% from the earliest to the later mummies. This is interpreted as a decrease in rainfall and increasing temperatures during that period, a reflection of the onset and continuing evolution of an arid tropical climate in ancient Egypt and North East Africa.

Oxygen stable isotope analysis is a common method of investigating individual human movements or migrations.^27^^,^^31^^,^^32^ In the body, the oxygen isotope composition of skeletal tissue is directly linked to that of oxygen consumed and this is controlled by δ^18^O of the local drinking water.^33^ This is related to the δ^18^O of local rainfall, which varies regionally depending on average local air temperature, distance from the source of water vapour and elevation above sea level. Consequently, variation in the isotopic composition of precipitation due to changes in climate and geography means that it is possible to identify migrant persons in a population.

However, factors such as dietary intake and individual physiology can affect these isotopic values and so oxygen analysis can be combined with strontium (^87^Sr/^86^Sr) isotope analysis for more accurate results.^34^^,^^35^^,^^36^^,^^37^ Strontium is taken up into enamel and bone during nutrition, and the geology of the local area affects the concentration of the strontium isotopes in the water and the plants grown there. As enamel formation is complete early in life and not replaced, it registers a strontium record of those early years. Again, this data can be compared to local environmental and geological parameters, helping to identify indigenous peoples from potential immigrants.

## Proteomics

Proteomics is the large-scale study of protein structure, function and interaction, and has applications in archaeology and in various biomedical disciplines, such as genetics, molecular biology, medicine and dentistry. Proteins in archaeological material, including teeth and dental calculus, are increasingly being explored following developments in high-throughput, high-resolution tandem mass spectrometry.^38^^,^^39^^,^^40^

Proteomic analysis of ancient dental calculus can reveal information about individual diets, including plant, animal and dairy product consumption. A proteomic study of calculus by Hendy and collaborators^41^ analysed data from 100 samples in England ranging from the Iron Age to the post-medieval period (800 BC - AD 1900). The results were able to elucidate foodstuffs, such as milk and meat, that are otherwise invisible by microscopic approaches. Such research is also able to enhance the detection of understudied vegetative crops, especially in regions where micro- and macro-botanical remains are poorly studied or not preserved.

## DNA

In today's world, DNA extraction and testing has many uses. It is a crucial component in criminal investigations, it can determine paternity and it is important in diagnosing medical conditions and in the development and creation of vaccines and hormones.

Genetic sequences have traditionally been generated solely from modern individuals. With the advent of ancient aDNA research some 35 years ago, the way in which scientists addressed an entire array of questions in anthropology, evolutionary biology and the environmental and archaeological sciences changed. aDNA extracted from human bone and teeth can potentially be used to determine the sex of an individual,^42^^,^^43^^,^^44^ indicate familial relationships,^45^^,^^46^^,^^47^ study population movements,^48^^,^^49^^,^^50^ provide phylogenetic information^51^^,^^52^ and identify the presence of disease pathogens.^53^^,^^54^^,^^55^

Teeth are frequently the only part of the body that survives in an archaeological setting and fortuitously, their hard, robust and chemically stable nature often permits the retrieval of aDNA. However, aDNA sampling of teeth, although optimised for efficient DNA extraction, is destructive, relying as it does on drilling, sectioning and powdering the sample. Far less destructive is the technique for extracting aDNA from dental cementum on the root surface of the tooth.^56^ The cellular cementum is rich in cementocytes and these DNA-containing cells remain encased in the mineral structure of the tooth after death. In addition, cementum contains a substantially higher proportion of endogenous DNA compared with dentine from the same tooth. Enamel is largely inorganic and contains very little DNA.^57^

The dental pulp is located in a closed cavity, well-protected from the environment by the enamel, dentine and cementum, which, being hard mineralised tissues, are resistant to degradation after death. Shielded as it is, ancient pulpal tissue is an ideal reservoir for the detection of blood infection through aDNA and protein identification.^58^ On a practical note, aDNA extraction from the dental pulp is a simpler process than from hard tissues, such as bone, as it does not require prior decalcification.^59^

Molar teeth are usually the teeth of choice as the volume of the pulp correlates with the amount of aDNA recovered. The pulp chamber can be accessed through the occlusal surface of the crown^60^ or via the root apex,^61^ before then sampling the tissue utilising endodontic instrumentation. Both the techniques minimise damage to the morphological structure of the tooth.

Molecular analysis of ancient pulpal tissue can help to determine the pathogens that were involved in historic diseases and epidemics. Plague epidemics have been estimated as killing millions of people over the millennia, but diagnosis has previously relied on ancient texts and historical clinical observations. The epidemic known as the ‘Black Death' or ‘plague', first occurring in the mid-1300s, eventually spreading to most European countries, is considered to have killed an estimated 17-28 million individuals.^62^

In 1998, Yersinia pestis bacteria was found for the first time in 300-year-old dental pulp specimens from people buried in documented plague cemeteries by the detection of DNA sequences.^59^ Although there was some controversy about these earlier results, more recent studies have confirmed the findings.^63^^,^^64^

A long-standing controversy in the historical record has been which pathogen was the cause of the ‘Plague of Athens' during the Peloponnesian War. This has remained a matter of debate among scientists who have had to rely exclusively on Thucydides' ancient descriptions, from which several possible diagnoses had been proposed. However, more recently, a mass burial pit unearthed in the Kerameikos ancient cemetery of Athens, and dating back to the time of the plague outbreak (around 430 BC), has been identified. The dental pulp of the individuals buried there was sampled and the aDNA results have been able to implicate typhoid fever as the probable cause of the plague.^65^

## Dental calculus analysis

**Fig. 2:**
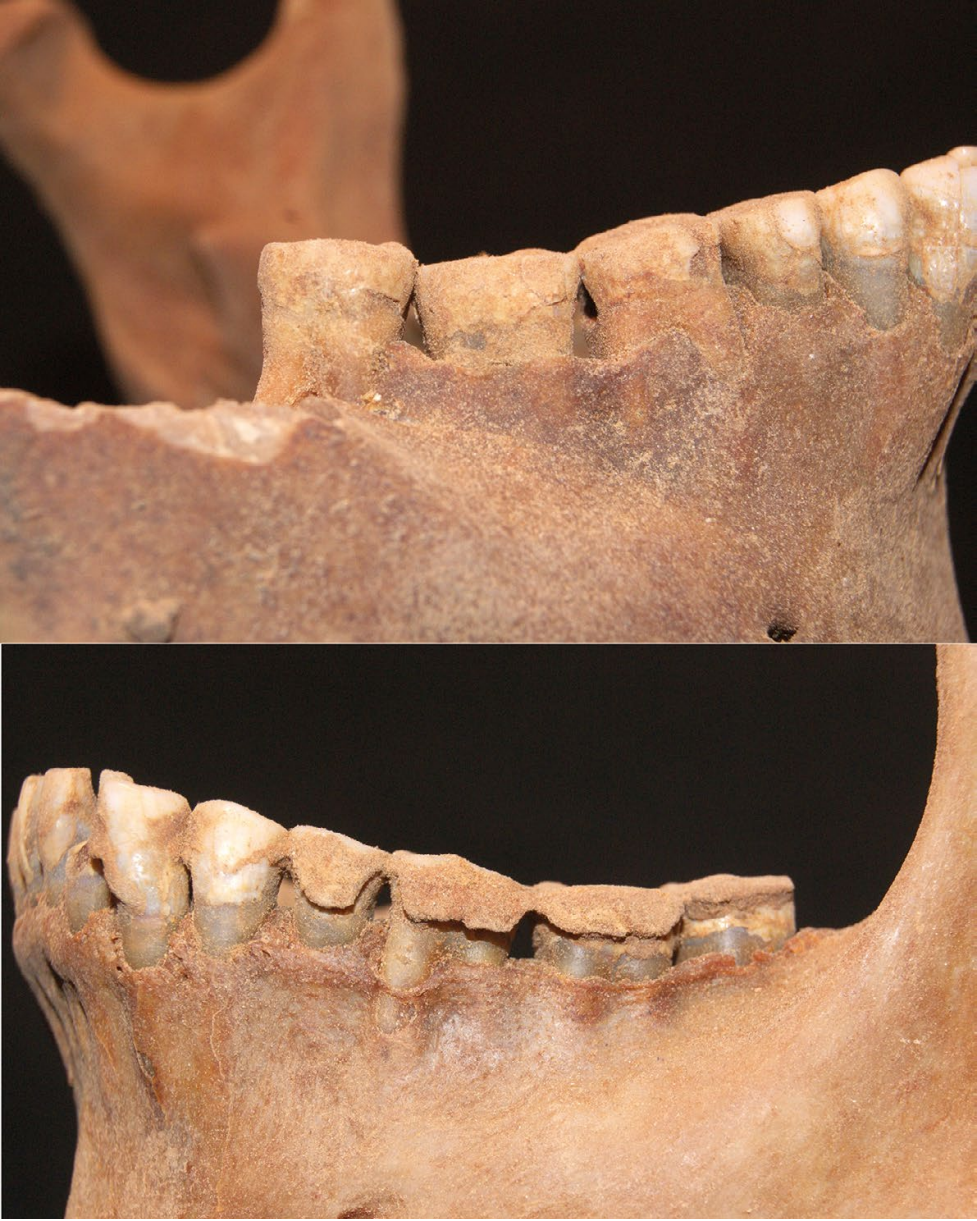
Gross calculus deposition on a male mandible from Theban Tomb 291 at Deir el-Medina, Egypt, c. 1350 BC. Reproduced with permission from Anne Austin

Dental calculus which develops via the mineralisation of plaque can remain for millennia on ancient skeletal remains, even as far back as the time the Neanderthals were alive^66^ (Fig. 2). Previous studies have demonstrated that calculus can preserve ancient biomolecules (DNA, proteins [see proteomics above], metabolites) and dietary microfossils (pollen, starch).^67^^,^^68^^,^^69^^,^^70^ Ancient calculus is recognised therefore as a repository of bio-information that can provide insights into diets and health in the past, as well as early oral microbial communities.

Through the study of environmental debris in dental calculus, not only can an overview of an individual's historical dietary habits be obtained, but information about the environment, individual behaviours and social culture changes can all be revealed^52^^,^^71^^,^^72^^,^^73^^,^^74^ (also see Forshaw^75^ for a review of this topic). The advantage of using calculus in dental analyses is that it is not an inherent part of the skeleton but a secondary material and thus may overcome curatorial concerns regarding the destructive analysis of primary biomaterials such as teeth.^76^

## Accentuated growth lines

Mineralised tissues, such as bone, enamel, dentine and cementum preserve regular incremental lines in their microstructure that are related to physiological cycles.^77^ Similarly, physiological stressors such as birth, weaning, malnutrition, disease, climate and lifestyle changes impact normal matrix formation and cause aberrant growth lines.^78^^,^^79^ However, primary secretions in enamel and dentine cease when the tooth is formed, so they are not useful for investigating later-life history.

**Fig. 3:**
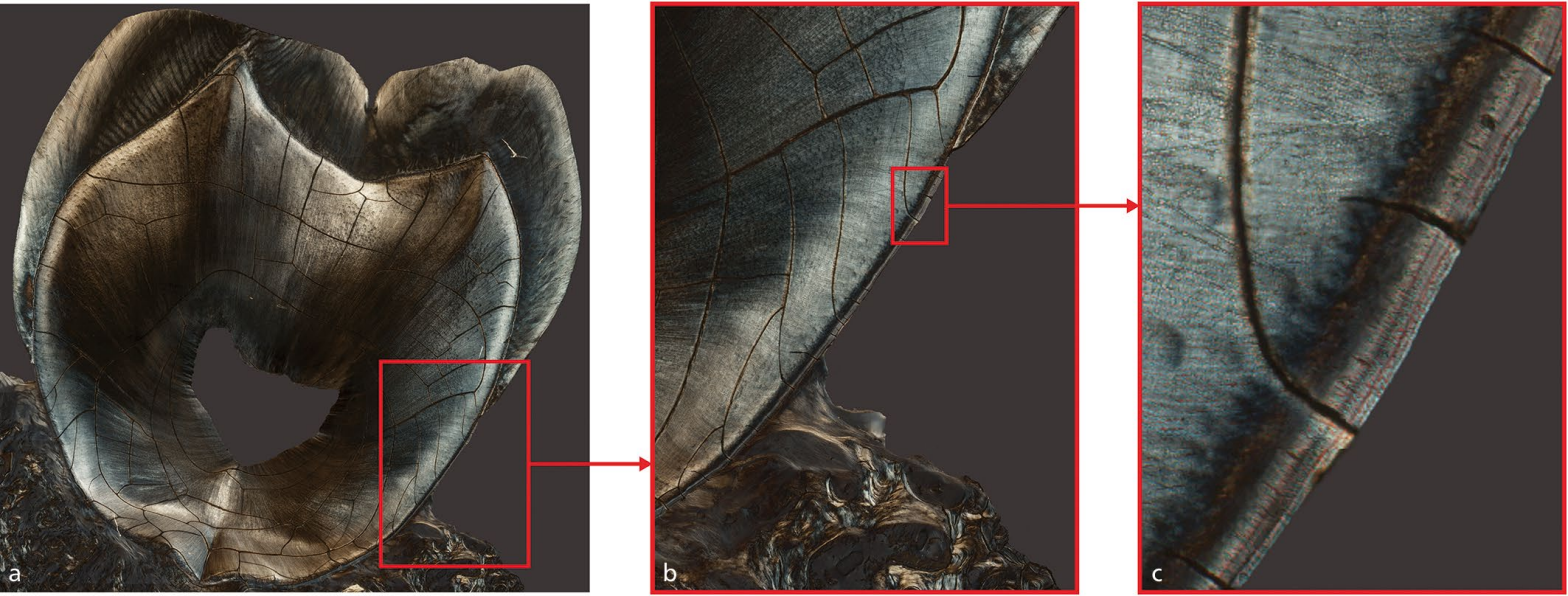
Cementum: X and Y planes of micrographs obtained in polarised light of a longitudinal section of a maxillary second molar. a) Whole tooth section. b) Magnification of panel A. c) Magnification of panel B. To the left is the dentine, covered by the layers of cementum; three distinct cementum bands are visible. Reprinted from P. Cerrito *et al*., ‘Parturitions, menopause and other physiological stressors are recorded in dental cementum microstructure', *Scientific Reports*, 2020.^81^ Licensed under a Creative Commons Attribution 4.0 International Licence

Dental cementum is the only incrementally growing mineralised tissue that is deposited throughout an individual's life and is not subject to remodelling.^57^ Consequently, not only are early life events preserved, but processes such as parturition, menopause and diseases all create histological markers in the cementum. These physiologically demanding episodes create a narrower cementum growth layer as a consequence of a slower progression of the mineralisation front of the cementoblasts. With advances in histological techniques and digital technologies, such as real-time microscopic image recording, the growth lines in the cementum, which are visible as changes in the tissue refractive index, are now able to be analysed and correlated to such occurrences^80^ (Fig. 3). In addition, a novel analytical method has been described to infer the age at which the event occurred by the study of these abnormal lines in the cementum microstructure.^81^ However, histological analysis does not differentiate between the various events and for this, elemental analysis of the growth lines is needed.^82^

More recently, a new non-invasive method which can visualise dental microstructures has been developed (a 3D imaging technique - synchrotron x-ray microtomography). The imaging method is able to detect physiologically impactful events in the cementum at submicrometre resolutions without having to destructively section the tooth.^5^ The technique also shows promise as a future non-destructive, age-at-death estimation method but at present, it is considered that this latter application is still in its infancy and will require further development to improve and establish its viability.^83^

## Hormones

Cortisol is a common stress biomarker and has important regulatory functions in cardiovascular, metabolic and immunological systems.^84^ Typically, samples of saliva, urine or hair are used to study levels of this hormone and to understand endocrine disorders in general. However, this measurement will only detect free or unbound cortisol and multiple samples taken over time are needed to study chronic stresses. Traces of cortisol are incorporated into tooth structures, possibly by the blood stream or saliva, and recent studies have suggested that dental cortisol profiling is able to provide a more dependable indication of prolonged stress conditions.^85^ As cortisol is preserved within the tooth structure, then analysis of cortisol concentrations in ancient teeth can provide information on ‘stress experience', an indicator of health and wellbeing in the past.^86^ These investigations are in their early stages and future studies may be able to expand the research potential of this technique.

## Trace elements

Trace elements are minerals present in body tissues in minute quantities, usually less than 0.1% by volume. Many function primarily as catalysts in enzyme systems and have an important and complex role in metabolism.^87^ Both deficiency and excess of the elements can cause clinical effects. Teeth are suitable indicators of trace element levels in ancient skeletal material, as the high degree of mineralisation and crystallinity make teeth more resistant to diagenetic changes compared with bone.^88^^,^^89^ The monitoring of trace element status can provide a range of information, including environmental conditions,^90^ physiological information relating to diet, growth and reproduction^91^ and can be used to determine palaeo-deficiencies and toxicities.^92^

Barium and strontium are particularly sensitive indicators of trace element exposure. Enamel incorporates these elements during amelogenesis in proportion to their dietary abundance and hence to their local environmental levels. It then retains the original levels of the elements and so can provide a chemical signature of the geographic origins of individuals.^93^ It has long been accepted that fluoride, related to the trace element fluorine, plays an inhibitory role in the development of dental caries. There are some indications that other environmental trace elements, such as molybdenum, may also affect caries experience.^94^^,^^95^

## Conclusion

Teeth are disproportionately prevalent in archaeological sites and for many years, researchers have been investigating teeth and acquiring important information relating to the diet, health and lifestyle of our ancestors. With the more recent application of advanced scientific methods in the study of odontology, a wealth of information is now able to be obtained. These types of data might not otherwise be retrievable from the archaeological record and can help with a better understanding of earlier populations. Such diverse techniques are continuously being developed and enhanced and further methods and procedures can be expected in the future.
